# Meta-synthesis of fatigue symptom experience and influencing factors in post-stroke fatigue patients in a qualitative study

**DOI:** 10.3389/fneur.2026.1763079

**Published:** 2026-07-08

**Authors:** Yawei Yu, Yingyu Chen, Ziye Li, Yu Duan, Zhanghui Guo, Hong Guo

**Affiliations:** School of Nursing, Beijing University of Chinese Medicine, Beijing, China

**Keywords:** fatigue, meta-synthesis, post-stroke fatigue, qualitative research, stroke

## Abstract

**Introduction:**

Post-stroke fatigue (PSF) is a common, persistent, and disabling complication in stroke survivors that significantly impairs quality of life. While most existing studies have used quantitative methods, qualitative evidence concerning patients’ fatigue experiences and the perceptions and coping strategies of relevant stakeholders remains limited. This study aimed to synthesise the experiences of fatigue among patients with PSF based on the Theory of Unpleasant Symptoms and to explore the perceptions and attitudes of patients, caregivers, and healthcare professionals.

**Methods:**

A qualitative systematic literature review and meta-synthesis was conducted following JBI methodology. Nine databases, including PubMed, Web of Science, and CNKI, were searched from inception to 25 March 2025. Eligibility criteria were developed using the PICoS framework. Qualitative studies and mixed-methods studies with extractable qualitative data involving patients with PSF, caregivers, or healthcare professionals were included. Study quality was assessed using the JBI Critical Appraisal Checklist for Qualitative Research.

**Results:**

Twelve studies were included. The synthesis of 30 research findings generated four synthesised findings: (1) the fatigue symptom experience of patients with PSF is multidimensional; (2) the influencing factors are multifaceted and significantly affect patients’ coping attitudes; (3) awareness of PSF among patients, caregivers, and healthcare professionals needs to be improved, while rehabilitation needs remain unmet; and (4) PSF management primarily depends on patient self-management, with healthcare professionals and caregivers playing complementary roles.

**Discussion:**

PSF is characterized by a multidimensional symptom experience influenced by physiological, psychological, and situational factors. There is an urgent need to develop appropriate measurement tools, strengthen education, implement personalized management strategies, and increase caregiver involvement. PROSPERO (CRD420251037784).

**Systematic review registration:**

https://www.crd.york.ac.uk/PROSPERO/view/CRD420251037784.

## 1 Introduction

Stroke is a major global health challenge, with its prevalence increasing annually worldwide. It ranks as a leading cause of mortality and long-term disability globally (1), as well as in China (2). Post-stroke fatigue (PSF) is a multidimensional experience, encompassing motor-sensory, emotional, and cognitive aspects, characterized by the early onset of tiredness, listlessness, and boredom during physical or mental activities, which typically cannot be relieved by rest (3). It is one of the most common, persistent, and disabling complications in stroke patients (4), International systematic reviews indicate that the global prevalence of PSF is approximately 29 to 68%, affecting survivors regardless of their regional or cultural contexts (5). Its pathogenesis remains unclear, and it may be related to biological, psychosocial, behavioral, and environmental factors (3). PSF can occur at any stage of stroke rehabilitation, seriously affecting patients’ rehabilitation progress and quality of life. In recent years, existing studies on post-stroke fatigue have mostly been quantitative. There is insufficient evidence exploring the feelings, cognition, and coping strategies related to post-stroke fatigue from the perspectives of PSF patients, caregivers, and health professionals. Existing qualitative studies (6, 7) have primarily focused on patients’ perceptions of fatigue, experiences, and coping strategies, exploring the subjective experiences of PSF patients regarding fatigue symptoms and their management processes (7), which also involve factors influencing PSF. However, the sample sizes of the above studies are small, and there has been no systematic induction and sorting of the symptom experiences, influencing factors, and symptom management strategies of PSF patients. The research results are relatively fragmented and have certain subjectivity.

The Theory of Unpleasant Symptoms (TOUS) emphasizes individual subjective experiences, helps understand the physical and psychological responses of patients when facing uncomfortable symptoms, and is one of the commonly used middle-range nursing theories in symptom research (8). By integrating the influencing factors of disease symptoms (including physiologic factors, psychologic factors, and situational factors), dimensions of symptom experience (intensity, distress, timing, and quality), and symptom performances, this theory has been widely applied to the assessment and management of chronic disease symptoms (9) as well as patients’ physical and psychological experiences when confronting unpleasant symptoms. Therefore, based on the TOUS, this study intends to conduct a meta-synthesis of qualitative studies, analyze the intensity, distress, timing, and quality dimensions of PSF patients’ experiences of fatigue symptoms, summarize the influencing factors of fatigue symptoms in PSF patients into physiologic, psychologic, and situational factors, and further sort out the cognition and attitudes of PSF patients, caregivers, and medical staff towards post-stroke fatigue symptoms. The aim is to gain an in-depth understanding of the unpleasant symptoms of post-stroke fatigue and provide a reference for PSF patients, caregivers, and health professionals to understand post-stroke fatigue further and optimize symptom management strategies.

## 2 Methods

The meta-synthesis approach was employed for design, aiming to systematically review, meta-aggregate, integrate, and interpret evidence from multiple published qualitative research sources. The review protocol had been published on the PROSPERO website (CRD420251037784).

### 2.1 Search methods and strategy

#### 2.1.1 Literature search

Two researchers systematically searched nine databases (PubMed, Web of Science, Embase, CINAHL, Cochrane Library, CNKI, Wanfang Data, VIP Database, and the CBM) using combinations of the keywords “Stroke” “fatigue” “post-stroke fatigue” “Psychological/Experience/Needs/perception*/feeling*” “qualitative research,” covering publications from database inception to March 2025. Search strategies were adapted for each database (Appendix 1: Search Strategy Table).

#### 2.1.2 Inclusion and exclusion criteria

In this study, the inclusion criteria for literature were set by the PICoS model. ① Population (P): Patients diagnosed with post-stroke fatigue, caregivers (including family members and friends of patients), and healthcare providers. ② Interest of Phenomena (I): Cognition, experiences, feelings, attitudes towards post-stroke fatigue symptoms among post-stroke fatigue patients, their caregivers, and healthcare providers, as well as influencing factors of fatigue symptoms in post-stroke fatigue patients. ③ Context (Co): Periods during which post-stroke fatigue patients receive rehabilitation in hospitals, rehabilitation centers, communities, or at home. ④ Study (S): Qualitative studies, including phenomenological studies and grounded theory studies; or extractable qualitative components from mixed-methods studies.

Exclusion criteria: ① Duplicate publications; ② Literature with incomplete information; ③ the full text is unavailable; ④ not publicated in Chinese or English; ⑤ with a quality rating of Grade C.

### 2.2 Study selection and quality appraisal

All identified citations were uploaded into Noteexpress, and duplicates were removed. Initially, two researchers independently screened article titles and abstracts against the inclusion and exclusion criteria. Potentially relevant articles proceeded to full-text review, where the same selection criteria were applied. Disagreements were resolved through discussion with a third researcher.

Two researchers evaluated the quality of the screened study using the JBI Critical Appraisal Checklist for Qualitative Research (2016) (10). Study that met all evaluation criteria was rated as Grade A, while those meeting partial criteria were rated as Grade B, and those failing to meet any criteria were rated as Grade C. In case of disagreements, a third researcher was consulted for further discussion and final evaluation. Ultimately, only study rated as Grade A or B was included, while those rated as Grade C were excluded (Table 1).

**Table 1:** Methodological quality evaluation of included literature (*n* = 12).

Study	①	②	③	④	⑤	⑥	⑦	⑧	⑨	⑩	Quality grading
Shan Cui	Y	Y	Y	Y	Y	N	N	Y	Y	Y	B
Yumeng Si	Y	Y	Y	Y	Y	N	N	Y	Y	Y	B
Ablewhite J	Y	Y	Y	Y	Y	N	N	Y	Y	Y	B
Alahmari WS	Y	Y	Y	Y	Y	N	N	Y	Y	Y	B
Alahmari WS	Y	Y	Y	Y	Y	N	N	Y	Y	Y	B
Delbridge A	Y	Y	Y	Y	Y	N	N	Y	Y	Y	B
Kirkevold M	Y	Y	Y	Y	Y	N	N	Y	Y	Y	B
White JH	Y	Y	Y	Y	Y	N	N	Y	Y	Y	B
Young CA	Y	Y	Y	Y	Y	N	N	Y	Y	Y	B
Bicknell ED	Y	Y	Y	Y	Y	N	N	Y	Y	Y	B
Eriksson G	Y	Y	Y	Y	Y	N	N	Y	Y	Y	B
Jacobi M	Y	Y	Y	Y	Y	N	N	Y	Y	Y	B

① Is there congruity between the stated philosophical perspective and the research methodology? ② Is there congruity between the research methodology and the research question or objective? ③ Is there congruity between the research methodology and the methods used to collect data? ④ Is there congruity between the research methodology and the representation and analysis of data? ⑤ Is there congruity between the research methodology and the interpretation of results? ⑥ Is there a statement locating the researcher culturally or theoretically? ⑦ Is the influence of the researcher on the research, and vice- versa, addressed?⑧Are participants, and their voices, adequately represented? ⑨ Is the research ethical according to current criteria or for recent studies, and is there evidence of ethical approval by an appropriate body? ⑩ Do the conclusions drawn in the research report flow from the analysis, or interpretation, of the data?

### 2.3 Data abstraction

Data were extracted from included studies, focusing on author, country, year of publication, participants, qualitative research methods, interesting phenomena, contextual factors, results (Table 2 for details).

**Table 2:** The basic characteristics of the included literature (*n* = 12).

Author/country/year	Participants	Methods	Interesting phenomena	Contextual factors	Results
Shan Cui China, 2024	PSF patients (*n* = 16)	Phenomenological research method; Semi-structured interview; Colaizzi’s seven-step analysis method	The feelings and coping experiences of stroke patients towards post-stroke fatigue.	NR	The perceived characteristics of post-stroke fatigue; the coping styles of post-stroke fatigue.
Yumeng Si China, 2024	PSF patients (*n* = 16)	Phenomenological research method; Semi-structured interview; Thematic Analysis	The fatigue experience and coping strategies of patients with post-stroke fatigue.	NR	The fatigue experience of patients with post-stroke fatigue; fatigue coping strategies
Ablewhite J UK, 2022	PSF patients (*n* = 20); Caregivers (*n* = 8)	Phenomenological research method; Semi-structured interview; Framework Analysis	How stroke survivors and their caregivers manage post-stroke fatigue.	Due to the COVID-19, all interviews were conducted by telephone.	Most participants had found their own ways of coping and their personal strategies included acceptance of having fatigue; ‘pacing’ (spreading activities out and interspersing with rest periods); keeping a diary in order to plan activities and to identify ‘trigger’ activities which induced fatigue; talking to (and educating) others about having fatigue; using relaxation; and accessing professional advice and support. The burden placed on caregivers was considerable and they often had to oversee the post-stroke fatigue management strategies used.
Alahmari WS Saudi Arabia, 2023	Physiotherapists (*n* = 8); Occupational therapists (*n* = 8); Physiatrists (*n* = 8)	Phenomenological research method; Semi-structured interview; Thematic Analysis	Healthcare professionals’ understanding of post-stroke fatigue; their perspectives and experiences regarding the management of post-stroke fatigue.	A meeting room in hospital	Five overarching themes encompassing various subthemes and sub-subthemes were generated: ‘knowledge about post-stroke fatigue’, ‘diagnosing post-stroke fatigue’, ‘treatment approach’, ‘lack of awareness about post-stroke fatigue’, and ‘domains to improve’. The data indicated that participants used various strategies to manage PSF, including dietary changes, sleep hygiene, exercise, and energy conservation.
Alahmari WS Saudi Arabia, 2023	Adult patients (≥18 years old) diagnosed with cerebrovascular stroke (*n* = 8)	Descriptive qualitative research; Semi-structured interview; Thematic Analysis	Stroke survivors’ perspectives and experiences regarding post-stroke fatigue and its causes, the impact of fatigue on their daily lives, their coping strategies, and their views on the support provided by doctors and nurses.	A meeting room in hospital	Five main themes and several subthemes were generated: (1) description of PSF as both a physical and psychological experience; (2) perceived causes of PSF (physical causes, psychological causes, and stroke-related causes); (3) impact of PSF on daily life (activities of daily living and social interactions); (4) coping strategies for PSF (pacing, self-motivation, and social participation); and (5) perspectives about support from caregivers and healthcare practitioners (lack of support, provision of information about PSF and its management, physiotherapy interventions, encouragement, and overprotectiveness).
Delbridge A Australia, 2023	PSF patients (*n* = 17); Caregivers (*n* = 8)	Semi-structured interview; Thematic Analysis	The experiences of patients with post-stroke fatigue; the management strategies of patients and their caregivers.	Audio and/or video recorded	We identified four themes: (1) fatigue is unexpected after stroke and symptoms vary; (2) the individual experience of fatigue is complex, influenced by multifactorial and biopsychosocial factors; (3) learning to adapt and accept fatigue; and (4) Strategies to manage fatigue and personal approaches to rest.
Kirkevold M Norway, 2012	Stroke patients (*n* = 32)	Grounded Theory; Interview Method	How stroke survivors experience fatigue, how they understand and manage it, and how fatigue affects their daily lives.	NR	Patients clearly described and differentiated their experience between: (1) tiredness as an ordinary life event and (2) fatigue as a post stroke life condition. Three fatigue-transforming strategies were identified, being on a mission, settling for less and stalling. Stalling seemed to put the stroke survivors in a particularly vulnerable situation. Over time, some participants moved between these two tiredness/fatigue manifestations and their range of strategies.
White JH Australia, 2012	PSF patients (*n* = 31)	Grounded Theory; Semi-structured interview	Explore the fatigue experienced by community-dwelling stroke survivors within 1 year after stroke, and the relationship between fatigue and emotional disorders.	NR	Three trajectories emerged regarding the participants’ experiences of fatigue including experience of fatigue, coping strategies and knowledge.
Young CA UK, 2013	PSF patients (*n* = 10)	Interpretative Phenomenological Analysis; Semi-structured interview	Explore the experience of post-stroke fatigue in stroke patients and its subjective impact on them.	A quiet room in hospital	Six main themes were identified. Tiredness/sleep was recognized in all the narratives, and themes of restriction, frustration, and determination/coping reflected varying degrees of physical, cognitive, and psychological dimensions to fatigue. Depression/motivation was also identified, reflecting low mood and helplessness. The remaining theme support indicated a social dimension, with patients recognizing the need for professional and familial support. Further subthemes were identified., and the thematic descriptions of the physical and psychosocial aspects indicated the complexity of fatigue and unique patient profiles. A holistic overview of each narrative furthered an understanding of the dynamic interrelationships between these aspects and their impact on the patient. There were prevalent patterns, but these were different for each patient.
Bicknell ED Australia, 2022	PSF patients (*n* = 14); Caregivers (*n* = 9)	Phenomenological research method; Semi-structured interview; Colaizzi’s seven-step analysis method	The experiences of patients with post-stroke fatigue during outpatient rehabilitation, including the perspectives of caregivers.	A private room at the facility or participant’s homes	Six themes were identified: (1) The unpredictable and unprepared uncovering of fatigue; (2) Experience and adjustment are personal; (3) Being responsible for self-managing fatigue; (4) The complex juggle of outpatient stroke rehabilitation with fatigue; (5) Learning about fatigue is a self-directed problem-solving experience; (6) Family and carers can support or constrain managing fatigue.
Eriksson G Sweden, 2023	PSF patients (*n* = 9)	Semi-structured Interview; Qualitative Content Analysis	Explore the ways in which individuals experience and cope with fatigue in daily activities and social interactions within 5 years after a stroke.	In participants’ homes or at their workplace	Handling post-stroke fatigue—a long slow process with invisible adjustments in everyday life; post-stroke fatigue– a sudden change felt in body and mind; Living life peacefully and at a slower pace; Change or stop doing – a way to handle complex activities; Support and strategies improve everyday life; Strategies and daily routines make PSF less noticed.
Jacobi M Amsterdam, 2024	PSF patients (*n* = 10); Health professionals (*n* = 12)	Focus group interview; Semi-structured Interview; Reflexive Thematic Analysis	Stroke patients and healthcare professionals’ experiences and perspectives on guidelines for post-stroke fatigue	Due to the COVID-19, all interviews were conducted online	Three themes were identified. Guidance in fatigue management did not always match the needs of people/patients with stroke. Professionals were positive about the provided fatigue guidance (e.g., advice on activity pacing), but found it could be better tailored to the situation of people/patients with stroke. Professionals believe the right time for post-stroke fatigue guidance is when people/patients with stroke are motivated to change physical activity behaviour to manage fatigue – mostly several months after stroke – while people/patients with stroke preferred information on post-stroke fatigue well before discharge. Follow-up care and suggestions for improvement described that follow-up support after rehabilitation by a stroke coach is not implemented nationwide, while people/patients with stroke and professionals expressed a need for it.

### 2.4 Data synthesis

**Figure 1:**
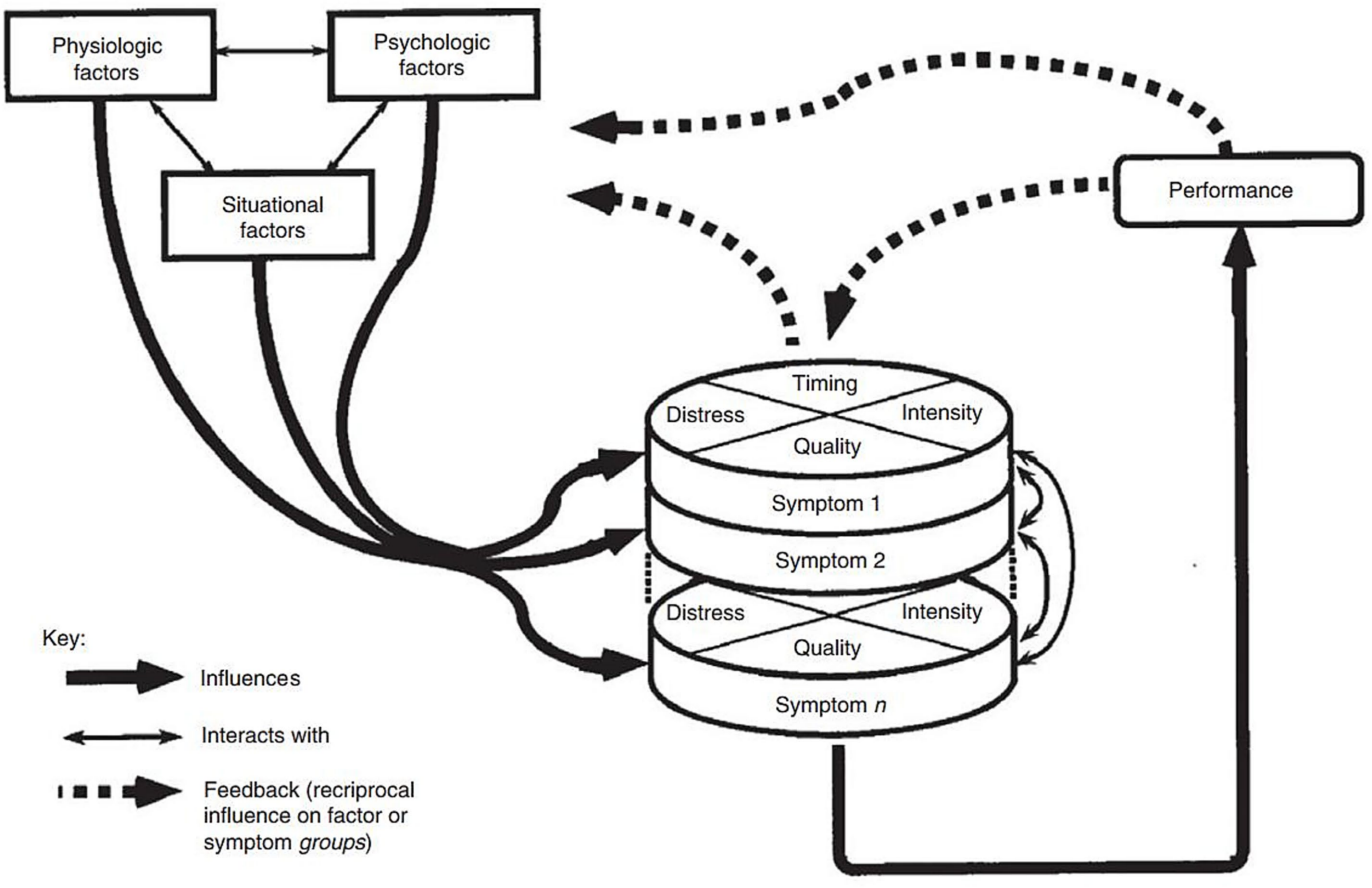
The model of the theory of unpleasant symptoms.

A meta-synthesis approach following the JBI meta-aggregation method (11) was employed to synthesize the included qualitative data. Meta-aggregation, as defined by JBI, refers to the process of aggregating findings from qualitative studies into categories and synthesized findings without re-interpreting the primary data. In this study, a ‘finding’ was defined as a verbatim extracted theme, sub-theme, or category supported by participant quotes and textual illustrations from the results or findings sections of the included primary studies. Two reviewers independently extracted and synthesized data. Under the premise of understanding the philosophical theories and methodologies underpinning qualitative research, the researchers iteratively read and cross-compared the findings to identify thematic relationships. Similar results were grouped into preliminary categories, which were further analyzed for interrelationships to form synthesized themes. These themes were then refined through critical interpretation and theoretical contextualization to generate novel insights. Discrepancies between the two reviewers were resolved through discussion until consensus was reached; if disagreement persisted, a third independent reviewer was consulted to arbitrate and finalize decisions (Figure 1).

### 2.5 Assessment of confidence in the review findings

Based on the ConQual system (12), the quality of the synthesized results was evaluated from 5 aspects of dependability and three aspects of credibility. This system classifies the quality of the evidence body into four levels: high, moderate, low, and very low.

## 3 Results

### 3.1 Characteristics of included studies

**Figure 2:**
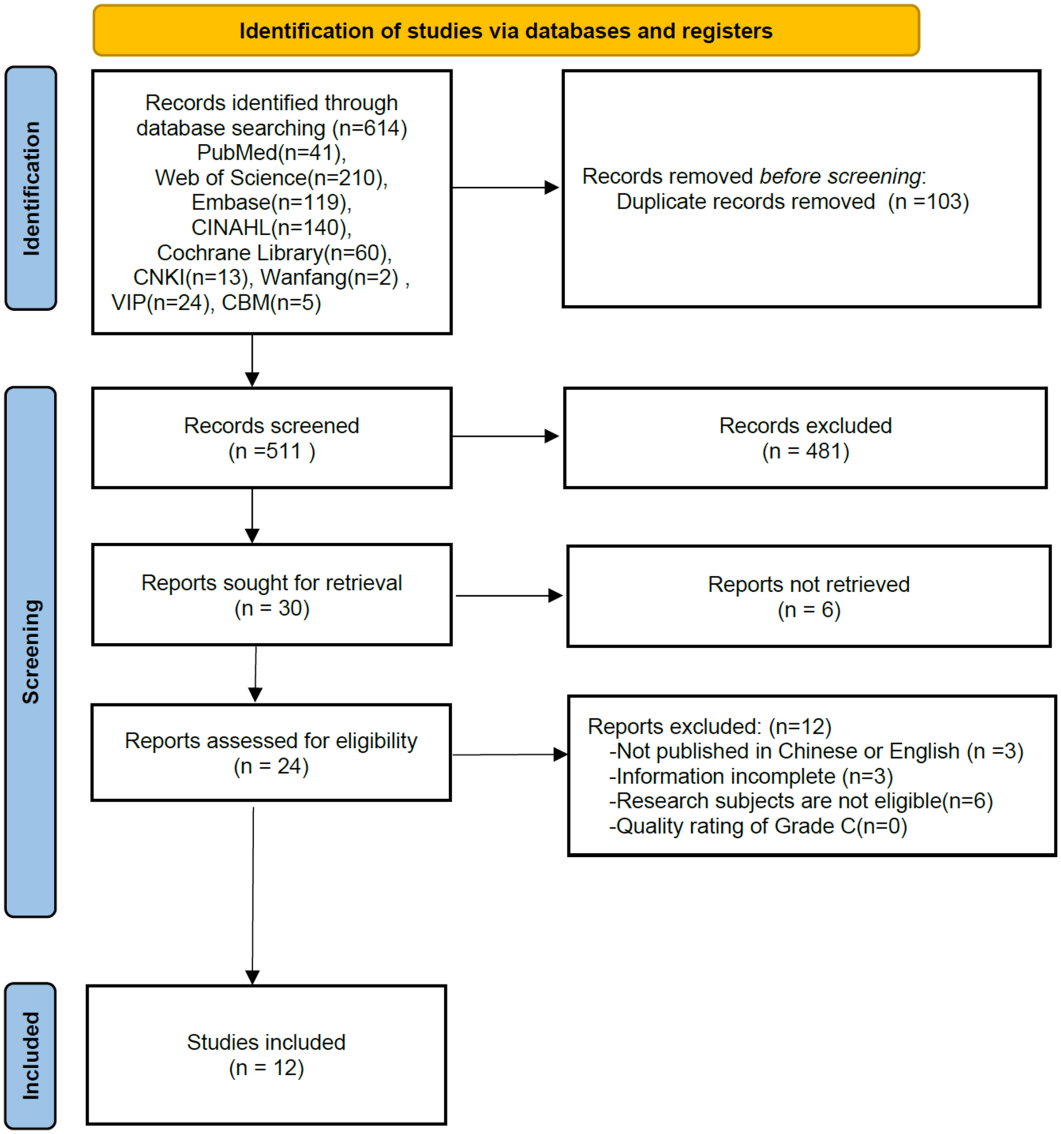
PRISMA diagram of the literature search.

A preliminary search yielded 614 articles, and 511 articles remained after duplicates were removed. Through reading and screening by the inclusion and exclusion criteria, 12 articles were finally included (6, 7, 13–22). The detailed selection process is shown in Figure 2 (PRISMA flowchart).

The 12 articles included in the meta-synthesis involved a total of 183 stroke patients, 25 caregivers, and 36 healthcare providers. The basic characteristics of the included articles were presented in Table 2.

### 3.2 Methodological quality

A total of 12 studies were reviewed for methodological quality. The overall quality assessment results of all included articles were Grade B, as shown in Table 1. The main methodological limitations were the insufficient reporting of researcher reflexivity and the limited description of the relationship between researchers and participants. However, the included studies generally demonstrated consistency between research methodology, research questions, data collection methods, and data analysis processes, indicating an acceptable overall methodological quality.

### 3.3 Findings

**Figure 3:**
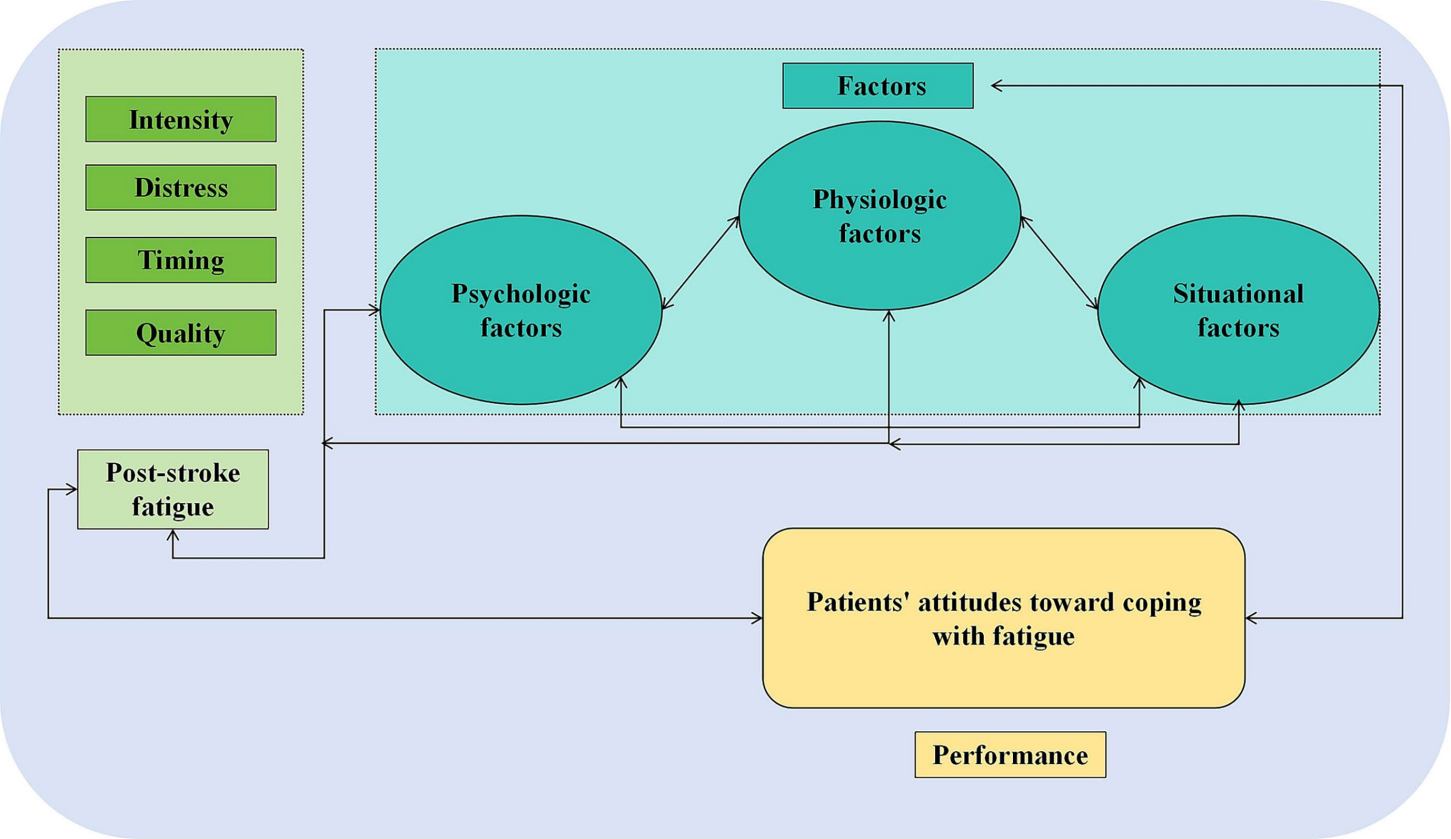
Synthesized findings.

Based on the Theory of Unpleasant Symptoms, the included data were synthesized using the meta-aggregative approach (11). From the 12 studies, 30 research findings were extracted, the number of findings extracted from each primary study ranged from 3 to 11. These 30 findings were then summarized into 12 categories and finally synthesized into 4 synthesized results. For details, see Table 3 and Figure 3.

**Table 3:** Overview of categories, synthesized categories and synthesized outcomes.

Synthesized findings	Synthesized categories	Categories
Synthesized finding 1: The experience of fatigue symptoms in post-stroke fatigue patients was multidimensional.	Category 1: Intensity dimension of symptoms	Outcome 1: Fatigue intensity was tolerable. Outcome 2: Fatigue intensity was unbearable.
	Category 2: Distress dimension of symptoms	Outcome 3: Physical distress Outcome 4: Psychological distress Outcome 5: Behavioral distress
	Category 3: Timing dimension of symptoms	Outcome 6: The timing of PSF onset Outcome 7: PSF persistence
	Category 4: Quality dimension of symptoms	Outcome 8: Physical fatigue Outcome 9: Psychological fatigue Outcome 10: Cognitive fatigue
Synthesized finding 2: The influencing factors of post-stroke fatigue were multidimensional and are counteracted by fatigue, affecting patients’ attitudes toward coping with fatigue.	Category 5: Physiologic factors	Outcome 11: Demographic characteristics Outcome 12: Physical function
	Category 6: Psychologic factors	Outcome 13: Negative emotions and coping attitudes Outcome 14: Positive emotions and coping attitudes
	Category 7: Environmental factors	Outcome 15: Living environment Outcome 16: Social environment
Synthesized finding 3: The cognition of PSF among patients, caregivers, and healthcare professionals needed improvement, and the rehabilitation needs of PSF patients remained unmet.	Category 8: Current status of cognition on post-stroke fatigue	Outcome 17: Patients lacked cognition of PSF Outcome 18: Caregivers lacked cognition of PSF Outcome 19: Health professionals lacked attention of PSF
	Category 9: Patients’ rehabilitation needs	Outcome 20: Needs of caregivers Outcome 21: Needs of healthcare professionals Outcome 22: Needs of peers
Synthesized finding 4: The management of post-stroke fatigue was primarily based on patients’ self-management, supplemented by symptom management assistance from healthcare professionals and caregivers.	Category 10: Self-management strategies	Outcome 23: Rest and sleep Outcome 24: Exercising Outcome 25: Planning Outcome 26: Pacing
	Category 11: Healthcare professionals’ management strategies and suggestions	Outcome 27: The management strategies provided by healthcare professionals Outcome 28: The timing of providing professional guidance
	Category 12: Caregivers’ assistance in symptom management	Outcome 29: The meaning of caregivers’ help Outcome 30: The states of caregivers’ involvement in fatigue management

#### 3.3.1 Synthesized finding 1

The experience of fatigue symptoms in post-stroke fatigue patients was multidimensional.

Based on the TOUS, this study examined the experience of fatigue symptoms in patients with PSF. The results showed that post-stroke fatigue was manifested in the following dimensions: the intensity dimension reflected patients’ tolerance of fatigue symptoms; the distress dimension included physical, psychological, and behavioral distress; the timing dimension involved the onset timing and persistence of symptoms; and the quality dimension encompassed physical, psychological, and cognitive fatigue.

Category 1: Intensity dimension of symptoms.

PSF patients perceived varying intensities of fatigue, which was reflected in their ability to tolerate fatigue. Some patients reported that the intensity of PSF was bearable during episodes [“…tiredness is a relative thing, and one constantly compares with ones earlier life…so in terms of that, relatively speaking, I am not at all tired, but I am tired compared to my previous life (18)”]. Fatigue occurred intermittently as an ordinary part of daily life (17) and became a routine life experience [“Fatigue is always present, and I feel a bit tired almost every day (6)”]. However, other patients stated that once post-stroke fatigue occurred, they felt helpless [“I just have to stop. I cannot actually continue I just have to stop (16)”], and some even could not perform self-care [“Sometimes, I feel too tired even just to eat my meal or go to the toilet (15)”]. In such cases, fatigue manifests as a persistent post-stroke life state, exerting a more profound impact on the lives of stroke survivors.

Category 2: Distress dimension of symptoms.

Fatigue symptoms caused multifaceted distress to PSF patients, threatening their physical and mental health. In terms of physical distress, fatigue limited patients’ physical activities [“My hands and legs do not obey me anymore; I get exhausted after a little movement (7)”], making it impossible for them to complete or sustain daily activities independently [“I feel exhausted even when eating or talking, and I’m tired all day long (7)”].

Regarding psychological distress, fatigue undermined patients’ independence in daily life, leaving them struggling to maintain a regular routine [“Sometimes I’m so tired that I do not want to wash my face, brush my teeth, or eat—I just want to lie down (6)”]. They had to rely on others’ assistance [“Now I feel like I cannot do anything; my family has to help me, and I feel useless (6)”]. Over time, however, patients lost a sense of control over their lives, accompanied by guilt from long-term dependence on others, which might trigger negative emotions such as anxiety, depression, and anger [“Suddenly, my whole body feels weak, my mood turns bad, and I become irritable. That’s how fatigue makes me—my temper flares up (7)”].

In terms of behavioral distress, fatigue disrupted patients’ daily lives. Before the stroke, patients often assumed multiple social roles in life and work, but the stroke suddenly paused their daily lives. During the long rehabilitation period, they could no longer fulfill their original social roles [“I used to do all the housework, but now I’m always exhausted and have no strength to work. My spouse has to buy groceries, cook, take care of the grandchildren, and look after me (7)”]. Many patients reported that they were no longer the same person after the stroke [“And you wanted to be just like you used to be, even though you felt that you were not (21)”]. Fatigue made self-care more difficult, let alone participating in housework [“I used to be very capable, but now I cannot do anything; even a little work at home leaves me with a sore back and waist (6)”].

Socially, some patients noted that fatigue limited their mobility and drained their energy significantly, hindering their participation in social activities (21). Recreational activities that had felt enjoyable and easy before the stroke had become more challenging [“Something that was originally fun can become difficult because you are too tired (21)”], and even conversations were restricted [“You cannot talk to people for long (19)”]. Patients still in work reported that their physical strength and energy were far weaker than before, making work tasks harder to complete [“Compared to before getting sick, my energy is insufficient—I get tired very easily, and work tasks are hard to carry out (6)”].

Category 3: Timing dimension of symptoms.

For PSF patients, the manifestation of fatigue symptoms might change over time, specifically in terms of the timing of symptom onset and symptom persistence. Most PSF patients reported that fatigue occurred after returning home [“I wasn’t immediately…showing signs that I was affected by fatigue… At the hospital I got a lot of rest…But then I came home and (it) came to the forefront a bit more (20)”]. Its onset was unpredictable and always came without warning [“Suddenly, I feel extremely fatigued, unable to do anything, even unable to walk, which has a great impact on me (7)”], catching PSF patients off guard [“You never know when it will come (20)”]. A few patients described experiencing persistent fatigue [“I feel a bit tired almost every day (18)”], and most reported that symptoms worsened during the day [“It is usually most severe at noon, not at night, and sometimes I feel dizzy and exhausted when I wake up in the morning (7)”].

Category 4: Quality dimension of symptoms.

The fatigue experiences of PSF patients often included three aspects: physical fatigue, psychological fatigue, and cognitive fatigue. Patients frequently used terms such as pain, weakness, sleepiness, loss of balance, lack of energy, headache, and tachycardia to describe their experience of PSF. In terms of physical fatigue, some patients reported unpleasant sensations such as pain and weakness [“I feel heartache, rapid pulse, tiredness, and weakness (15)”]. Fatigue not only deprived patients’ energy but also caused fluctuations in emotional states [“I have become quite irritable, especially easy to get anxious, and no one in the family dares to provoke me (6)”], leading to a loss of motivation in life and a significant decrease in enthusiasm and interest in daily activities [“I used to like square dancing, but I am too tired now and have stopped (6)”].

Additionally, fatigue impacted patients’ cognitive function to some extent, making it challenging for them to read and understood information. Patients could not concentrate [“Fatigue makes me unable to focus and easily distracted (15)”], struggling to complete tasks requiring high concentration such as reading and writing [“I have no energy to do the things I used to enjoy. I used to like reading, but now I read a few pages and do not know what it’s about (6)”].

#### 3.3.2 Synthesized finding 2

The influencing factors of post-stroke fatigue were multidimensional and are counteracted by fatigue, affecting patients’ attitudes toward coping with fatigue.

Based on the TOUS, this study summarized the influencing factors of fatigue symptoms in PSF patients into physiologic factors, psychologic factors, and situational factors. There was an interactive relationship between the influencing factors of each dimension and fatigue, which led to complex symptom experiences in PSF patients and further influenced their attitudes toward coping with fatigue. Among these, psychological factors were most closely related to the attitudes of PSF patients toward coping with fatigue. Physiologic and environmental factors could indirectly influence patients’ emotional states by affecting their perception of fatigue symptoms, ultimately shaping their collective attitudes toward coping with fatigue.

Category 5: Physiologic factors--Reciprocal influence with patients’ perception of fatigue symptoms.

Healthcare professionals noted that specific demographic characteristics, such as gender and age, [“Also, fatigue after a stroke depends on the patient’s lifestyle before the stroke, age and past medical history. All these factors affect the severity of fatigue after stroke. The physical activity level before a stroke affects or reflects the severity of the fatigue after the stroke.’ (14)”], as well as the presence of comorbidities like diabetes. Some rehabilitation therapists attributed PSF to physical factors, arguing that stroke sequelae, including symptoms like mobility impairment and debility, affected patients’ perception of fatigue (18). Patients with better physical function experienced lower levels of fatigue [“If a patient has a low functional independence level or functional independence score, of course he or she has more fatigue. Conversely, if a patient has a high functional independence level or functional independence score, of course he or she has less fatigue (14)”]. PSF patients also believed that long-term inactivity led to a decline in their energy levels, making them more susceptible to fatigue symptoms. Meanwhile, patients reported that their prolonged state of fatigue prevented them from regaining daily activity abilities, resulting in a decline in physical function (15).

Category 6: Psychologic factors--Interaction between PSF patients’ perception of fatigue and their emotional states, different emotional states influenced patients’ coping attitudes.

Patients with PSF perceived fatigue symptoms as closely intertwined with their emotional states, and different psychological states led patients to adopt distinct coping strategies for managing fatigue. Some patients perceived negative emotions as a direct cause of PSF and also recognized the reciprocal interaction between negative emotions and PSF (15). On the one hand, fatigue made it difficult for patients to return to daily life, and they felt that post-stroke fatigue and negative experiences had brought them stress and discomfort [“It’s hard to accept myself; I feel like I need to slap myself (6)”]. On the other hand, depressive emotions could trigger PSF [“I feel tired and exhausted, and this is caused by psychological factors (15)”]. The persistence of negative emotions affected patients’ physical and mental health, and the fear of fatigue could exacerbate fatigue [“Fear is pretty significant. Because I hit these wipe-out days … they are misery for me. Like they are low, as low, as low as a day can be for me (16)”].

Prolonged immersion in such negative emotional states made patients prone to an evasive mindset, unwilling to face real-life problems [“As long asI’m not dying, I do not want to deal with this issue anymore (6)”]. However, some patients indicated that evasion was not their intention. For them, fatigue often threatened their coping efforts (17). They strove to accept their current situation and adopt specific coping methods but seemed to struggle to find effective ways [“I’d very much like to do everything at once…I have…in my head all the things that I’d like to do…and it really tires me that I do not get to do them…because I’d so much like to sew, knit, crochet, make mosaics and a lot of other things…exercise and so on (17)”]. Due to the long-term physical and mental exhaustion from symptom distress, patients became overwhelmed by excessive self-blame and self-pressure, ultimately choosing inaction.

In contrast, positive psychological states and coping styles were beneficial for PSF patients, manifesting mainly in two approaches. Some patients were unwilling to wallow in their current situation and thus regarded coping with fatigue as a challenge [“I’ll try to set goals, because that’s what I’m like (19)”]. They often pushed themselves with a sense of mission. Despite feeling tired, many patients were determined not to succumb to this sensation [“I push myself to go out…I will not give in to it. I’m quite determined that if I want something, there’s a way around it; I never really give up (19)”]. They often motivated themselves to reach their goals [“I’ll try to set goals, because that’s what I’m like (19)”]. Sometimes they pushed themselves to their limits [“Even if I’m tired, I force myself to do it, gradually overcoming it (6)”] to advance the rehabilitation process. However, if patients always tried to push themselves excessively, the effect might be counterproductive (13).

Another perspective held that adaptation and acceptance of PSF were considered key to moving forward. Some patients stated that accepting the need to learn to live with PSF was crucial (13) [“Acceptance is important. You have to accept your current situation; not accepting it will not help and will only make things worse (6)”]. Accepting the persistence of fatigue and changes in abilities was key to gaining control over fatigue and establishing a new outlook on life. Some patients acknowledged that they could no longer live as they used to, were willing to accept fatigue as a regular part of life, and believed they needed to adjust their daily habits to adapt to it (17). For example, some patients, upon sensing tiredness, chose short rests and rejoined after regaining energy [“Listening to your body is really important. If you feel fatigued you need to stop and rest (16)”]. They perceived the ability to choose when to participate in or withdraw from activities as a source of self-control (17), which helped them better manage fatigue symptoms.

Category 7: Environmental factors--the change of situational factors influenced patients’ perception of fatigue.

PSF patients stated that due to the limitations of stroke sequelae, their lifestyle had become simpler and calmer than before the stroke. They realized that they were less likely to perceive tiredness when in a quiet environment [“If it was quiet at home, and the radio was not on, it was easier to concentrate and easier to learn new things (21)”]. Therefore, some patients tended to reduce interference factors in their living environment; they chose to move from cities to rural areas [“I think it was a rescue that I moved out here. So, I can kind of get this peace of mind. I mean, there’s no traffic or anything here (21)”], which reduced the unpleasant experience of PSF. In addition, living a calmer life meant they needed to reduce decision-making in their daily lives to avoid stress and distress caused by making decisions. Some patients moved from their original residences to apartments, believing that this reduced the number of daily decisions they had to make. They no longer had to worry about deciding on various trivial matters (such as snow sweeping, gardening, etc.).

The social environment was also a significant factor influencing fatigue symptoms in PSF patients, depending on the people included in that environment. On the one hand, unfamiliar social environments can cause patients to feel anxious, which can easily induce fatigue (16). For example, some patients reported feeling distressed while shopping [“I tried to go to the shop at times when there are few people there, I get so tired in the head, it feels completely empty and then I get a headache. There will be so much noise. There will be a lot of people and a lot of noise (21)”]. Contacting a large number of strangers in a short time was a challenge for patients. They needed to plan the shopping process [such as what to buy, how to get to the store, finding goods, paying, bagging, transporting goods home, etc. (21)] and make decisions during implementation. This contradicted their original intention of reducing daily decision-making, leaving them feeling tired. On the other hand, interacting with familiar people in a familiar social environment could reduce patients’ anxiety and help alleviate fatigue (16). Some patients stated that social activities (such as spending time with friends and attending social gatherings) helped distract them from fatigue symptoms, alleviated their experience of unpleasant symptoms, and brought about positive emotional states (19).

#### 3.3.3 Synthesized finding 3

The cognition of PSF among patients, caregivers, and healthcare professionals needed improvement, and the rehabilitation needs of PSF patients remained unmet.

Category 8: Current status of cognition on post-stroke fatigue.

Most PSF patients lacked cognition of post-stroke fatigue symptoms, considering PSF not an important health issue (14) and holding incorrect understandings of the symptoms [“I did not recognize it as post-stroke fatigue; I just thought it was due to old age (6)”]. The lack of relevant knowledge led to severe confusion about their fatigue experience [“I wanted to know why fatigue occurred; at first, I did not understand why I was so tired (7)”], and they were unprepared for the experience of fatigue and its impact on life. Additionally, healthcare providers stated that some patients were unaware of the existence of fatigue guidance, and the patients believed that the reason they did not receive such guidance was that they had not explicitly asked for help from healthcare professionals (22).

PSF patients found it challenging to describe their feelings, so they were often not understood or even misunderstood by caregivers [“Even if I said I was tired, no one was willing to listen, and I would be blamed (7)”]. Due to the lack of understanding of PSF among patients’ relatives and friends, their support for PSF patients was often absent (13), which negatively affected family roles and relationships [“Because my family could not see it, they did not understand my condition and called me lazy (6)”]. Caregivers’ neglect of patients’ fatigue symptoms led them to have unrealistic expectations for patients’ rehabilitation, which further exacerbated the patients’ suffering [“I think it is like a silent killer (20)”].

Furthermore, PSF patients indicated that healthcare providers did not pay sufficient attention to fatigue symptoms: they either “did not focus on it” (20), failed to mention it [“I told healthcare providers about my condition, and only then did they tell me it was post-stroke fatigue; no one had told me that post-stroke fatigue might occur (6)”], or attributed fatigue to other factors [“The doctor said my constant fatigue might be related to the medicine I was taking and that it was nothing serious (7)”]. Some patients mentioned that they had to take the initiative to consult professionals to obtain relevant guidance [“I felt it was more like I was searching for information myself rather than people providing it to me. But I wondered if we could get support if we did not seek help (13)”] and believed that the support obtained through the medical system was limited (21). Healthcare professionals also stated that their own understanding of post-stroke fatigue was insufficient [“To be honest, no, it PSF is not something that’s at the top of our most-important list because with a stroke survivor, we would all just go for the physical impairments (14)”]. They needed to learn more about how to assess such fatigue [“Maybe we need to ask about the outcome measures of fatigue after stroke, or read about how we can measure it. This is very important (14)”] and believed that medical practitioners needed significant improvements in diagnosing and subsequently managing post-stroke fatigue.

Category 9: Patients’ rehabilitation needs.

The rehabilitation needs of PSF patients were specifically reflected in their needs for support from caregivers, healthcare professionals, and peers. PSF patients desired attention and support from family members [“I hoped my daughter would take more care of me in the hospital. Sometimes when I was tired and did not even want to move in bed, it would have been good if she could get me a glass of water (7)”]. Talking with relatives and friends about their fatigue experiences had positive significance [“…just talking about it really helps people. And I think maybe it does not get always talked about. And then I think what’s really important is family and friends knowing about it as well (13)”]. In terms of needs for healthcare providers, PSF patients longed for professional support [“I wished someone could tell me more about how to deal with this (7)”], and they believed they should receive professional guidance at the initial stage of PSF onset (21). Additionally, almost all patients urgently wanted to gain social and group recognition [“It’s different now. I get tired after working for a while. I hoped the company could understand me; I did not want to be like this (7)”] and desired acceptance and recognition from others. Due to the presence of fatigue, patients felt social discrimination and could only hide their fatigue (18). PSF patients explicitly expressed their need for peer support, as only fellow patients could truly understand and empathize with their fatigue symptoms [“We could only complain to each other. At least someone could understand how I felt, that I wasn’t just making a fuss (7)”].

#### 3.3.4 Synthesized finding 4

The management of post-stroke fatigue was primarily based on patients’ self-management, supplemented by symptom management assistance from healthcare professionals and caregivers.

Category 10: Self-management strategies.

Due to the lack of guidance from professionals, PSF patients primarily relied on self-management for managing fatigue symptoms. PSF patients believed that rest and sleep were an effective way to alleviate fatigue (“it was important to ‘rest well’ every day (18)”). Rest and sleep could be synthesized into their daily lives as pauses that they could lie down and do nothing (“When I am tired, I let my brain rest, think about nothing, and neither watch TV nor use my phone (6)”). However, excessive daytime sleep would affect patients’ nighttime sleep [“This will prevent you from sleeping at night (18)”]. Patients expressed different views on using exercise as a fatigue management strategy. Some patients reported that exercise helped them regain energy [“I exercise regularly; exercise is a good thing for me and makes me more energetic (6)”], thereby delaying the aggravation of fatigue. However, others felt nervous about exercise and physical activity, believing that “overexertion” might be detrimental to managing post-stroke fatigue (13). PSF patients indicated that planning was their most commonly used management strategy. They adjusted their daily activities by prioritizing pending tasks (16) [“I’m not going walking today, because I’m not up to it. Well, I’m very tired from what I did yesterday (18)”]. During the planning process, keeping a fatigue diary played a supporting role. A fatigue diary helped patients review and identify key events that triggered fatigue in daily activities [“I found the diary to be good. It does challenge you to think about what you do every day and then you can see when you have good days when you have bad days and what tends to be triggering the cognitive fatigue (13)”], allowing them to adjust their plans according to their needs.

Although prior planning and arrangement were recognized as effective strategies by PSF patients, due to the variability of fatigue, plans for daily life needed to remain flexible and adaptable (18), highlighting the importance of the pacing strategy. “Pacing”—spreading out activities with interspersed rest—was described as a strategy that helped manage fatigue (18). Many patients stated that if they had important activities to attend at a particular time of the day, they would try to reduce activity levels or rest during other times of the day [“I may not use it in the way I suggested, but if we are going out or meeting friends, or if we are doing something that night and know it might be a bit busy, then trying not to do too much during the day works (18)”], thereby avoiding fatigue. Therefore, combining the “pacing” strategy with planning could help patients manage fatigue better.

Category 11: Healthcare professionals’ management strategies and suggestions.

The management strategies provided by healthcare professionals coincided with patients’ self-management strategies in terms of rest and sleep, exercise, and the pacing strategy. They particularly emphasized the importance of the pacing strategy for patients to manage fatigue symptoms [“It definitely does not solve the fatigue. But it really does lessen the limitations it imposes (22)”]. In terms of exercise strategies, healthcare providers suggested that patients conduct endurance training progressively [“During physiotherapy sessions, we focus on treadmill, bike and aerobic exercise, gradually increasing the patient’s fitness level (14)”] and recommended respiratory training [“I usually recommend and teach them breathing exercises (14)”].

Additionally, healthcare professionals emphasized the importance of managing factors that influence fatigue. They believed that to effectively treat PSF, it was crucial to identify which symptoms were caused by other comorbidities [“We have to do some lab tests, such as the thyroid function test, complete the blood screening for haemoglobin, irons. You have to check for irons and vitamin D (14)”]. Malnutrition and insufficient hydration were also considered influencing factors of PSF [“The patients may not have sufficient nutrition and hydration. They need these to be able to meet their energy demand; not having these will have an impact on their fatigue (14)”]. A few doctors mentioned some unrecognized treatment methods, such as prescribing antidepressants for PSF patients [“To manage PSF, I think there is a need for antidepressants as they are better for fatigue. The patients can do more practice exercises (14)”] to maintain patients’ energy levels in the future.

Both healthcare professionals and PSF patients believed that professional guidance had a positive impact on PSF symptom management. However, they had not reached a consensus on the timing of providing such guidance. Healthcare providers stated that guidance should be offered after patients perceived the specific impact of fatigue on their lives [“I recognize that sometimes after rehabilitation, people/patients with stroke first have to actually face some problems before they are motivated for something, for a next step (22)”], so they suggested guiding within a few months of the rehabilitation period. However, PSF patients indicated that they hoped to receive more information about post-stroke fatigue at discharge to prepare in advance [“I think the health professional should have told me beforehand: madam, your body is doing quite well again, but remember, a stroke involves a lot more. You have a few tough weeks ahead. Lie down every afternoon. Either dose activities or take it easy. At that moment, I needed that (22)”].

Category 12: Caregivers’ assistance in symptom management.

Both PSF patients and professionals believed that the participation of relatives and friends in symptom management was important (13, 14). Caregivers currently hold two main views: encouragement and overprotection (15). Some patients stated that caregivers could encourage them to actively and independently manage symptoms and conduct rehabilitation training [“My brothers, sister and especially my husband support me (15)”], thereby improving their health and function. Regarding overprotection, some patients mentioned that caregivers would provide as much help as possible [“My family does not want me to feel tired, so they give me whatever I want (15)”] and prevent them from participating in activities that might cause physical fatigue.

### 3.4 Results of the quality evaluation of meta-synthesis evidence body

The quality evaluation results of the evidence body are shown in Table 4. Among the included original studies, the methodology was consistent with the research questions, objectives, data collection methods, and data analysis and presentation methods; however, none described the influence of the researchers on the studies. The credibility analysis indicated that the quality of the synthesized evidence body was high, and all synthesized conclusions were derived from multiple clear research findings. Ultimately, the synthesized evidence body was rated as moderate, which had specific reference value. In the future, it will be necessary to standardize the application of qualitative research methods further to improve the credibility of the results.

**Table 4:** Quality rating of synthesized evidence bodies.

Synthesized outcome	Dependability	Credibility	ConQual grade
1. The experience of fatigue symptoms in post-stroke fatigue patients was multidimensional	Down grade 1 level	No change	Moderate
2. The influencing factors of post-stroke fatigue were multidimensional and are counteracted by fatigue, affecting patients’ attitudes toward coping with fatigue	Down grade 1 level	No change	Moderate
3. The cognition of PSF among patients, caregivers, and healthcare professionals needed improvement, and the rehabilitation needs of PSF patients remained unmet	Down grade 1 level	No change	Moderate
4. The management of post-stroke fatigue was primarily based on patients’ self-management, supplemented by symptom management assistance from healthcare professionals and caregivers	Down grade 1 level	No change	Moderate

## 4 Discussion

### 4.1 The experience of PSF patients was complex and influenced by multiple factors; there is an urgent need to develop multidimensional evaluation tools to assess post-stroke fatigue symptoms comprehensively

PSF is a multidimensional motor, emotional, and cognitive experience that can occur at any stage of stroke rehabilitation. It is characterized by early feelings of tiredness, listlessness, and weariness during physical or mental activities, which usually do not improve with rest (23). As an unpleasant symptom, the complex experience of post-stroke fatigue manifests in various dimensions, including intensity, distress, timing, and quality, making it challenging to measure objectively (24). Screening and assessing the presence and severity of fatigue in stroke patients is a prerequisite for effective management. However, there is no clear unified standard for specific screening and assessment tools for PSF patients. Previous studies have primarily employed universal evaluation scales, such as the Fatigue Severity Scale (FSS), Fatigue Impact Scale (FIS), and Fatigue Assessment Scale (FAS). These scales had no advantages in measuring the multidimensional symptom experience of PSF and cannot assess the temporal characteristics of fatigue symptoms.

This study suggests that healthcare professionals should select scales that are appropriate for evaluating patients’ fatigue symptoms, taking into account factors such as the patient’s situation, the scale’s target population, and its validity (3). Meanwhile, it is recommended that healthcare professionals, in combination with the Theory of Unpleasant Symptoms, develop specific multidimensional assessment scales for the complex symptom experience of PSF to further accurately evaluate the intensity, distress, and quality of fatigue symptoms, and continuously monitor the manifestation of PSF at different time points and their changes over time. It is recommended that nurses or rehabilitation therapists perform dynamic assessments using multidimensional tools at key rehabilitation time points (e.g., hospital admission, pre-discharge, first return to home rehabilitation, return to work) to capture the trajectory of fatigue symptoms.

### 4.2 PSF patients were affected by multiple factors; healthcare professionals should identify these factors early and provide targeted interventions

The Theory of Unpleasant Symptoms highlights that influencing factors of symptoms encompass physiologic, psychologic, and situational factors. These factors can interact with each other and have bidirectional relationships with symptom experiences. Existing meta-analyses have shown (4, 25, 26) that factors such as gender, place of residence, diabetes, hyperlipidemia, previous stroke history, family dysfunction, NIHSS score, coronary heart disease, self-care ability, post-stroke sleep disorders, pain, anxiety, depression, and use of sedatives may be influencing factors of PSF. The above evidence indicates that factors from physiological, psychological, and situational dimensions all affect fatigue symptoms in PSF patients to varying degrees, which is consistent with the results of this study.

In this study, influencing factors in the physiologic dimension mainly included demographic factors and the patient’s health status. Currently, the impact of gender on acute-phase fatigue in stroke patients remains controversial, possibly because patients of different genders have differences in the perception or expression of fatigue symptoms, and women may be more sensitive to their discomfort (27). This suggests that, on the one hand, clinical staff should pay attention to the manifestations of fatigue in female patients and provide them with corresponding guidance and support. On the other hand, they should also focus on the fatigue experience of male patients and encourage them to express their feelings about unpleasant symptoms actively.

Physical health is the basis for reducing patients’ perception of fatigue. Studies have shown (28) that patients with higher Modified Rankin Scale (MRS) scores experienced more severe post-stroke fatigue. Patients with high MRS scores had severe limb dysfunction, and most of them chosen to rest in bed, lack of physical activity made patients feel fatigued (29). In addition, diseases such as diabetes, coronary heart disease, and hyperlipidemia can lead to varying degrees of decline in patients’ physical function, making them more likely to perceive fatigue. This reminds clinical practitioners to remain vigilant, continuously monitor and evaluate patients’ limb function and PSF levels while treating stroke, strengthen the treatment and care of patients’ comorbidities, and reduce the impact of related comorbidities on patients’ fatigue symptoms.

In this study, influencing factors in the psychologic dimension were mainly manifested in different emotional states, which affect patients’ attitudes towards coping with fatigue. After a stroke, patients who cannot accept physical disability tended to adopt avoidance or withdrawal to alleviate inner pain temporarily. However, long-term avoidance is not beneficial for disease treatment and rehabilitation (30); on the contrary, it can lead to negative emotions such as anxiety and depression, which are significantly related to post-stroke fatigue. Patients who actively cope with post-stroke life can take the initiative to seek external help and reasonably vent their emotions, which is more conducive to physical and mental health (31). Therefore, healthcare providers should promptly screen and evaluate patients’ negative emotions, provide comfort and encouragement through active inquiry and empathy, and guide them to adopt a positive attitude and methods to face the disease, thereby reducing patients’ fatigue levels.

In addition, the results of this study shown that post-stroke fatigue patients tended to be in quiet and familiar environments; noisy and unfamiliar living and social environments may further exacerbate patients’ fatigue experience. This suggests that relatives and friends of PSF patients should assist them in improving their living and social environments. This can be achieved by actively participating in and accompanying patients to complete medical visits and rehabilitation exercises, providing a familiar social environment, and helping patients mitigate the impact of environmental factors on fatigue symptoms.

Based on the analysis of influencing factors in these three dimensions, it is recommended that healthcare professionals summarize the influencing factors of fatigue symptoms in PSF patients based on TOUS, in order to clarify further the predictors of post-stroke fatigue symptoms in different dimensions and provide references for healthcare providers to formulate PSF prevention and management strategies.

### 4.3 The cognitive level of PSF among patients, caregivers, and healthcare professionals needed to be improved; it is necessary to strengthen cognitive education for all parties and further meet the rehabilitation needs of PSF patients

The results show that the cognition of PSF among patients, caregivers, and healthcare professionals was insufficient. It is necessary to strengthen fatigue cognitive education for both doctors and patients to enhance their ability to identify PSF. As communicators and guides of healthy behaviors, healthcare providers should enhance their understanding of PSF. Patients should actively express their fatigue experiences and rehabilitation needs, and take the initiative to seek help from healthcare providers and caregivers.

The rehabilitation needs of stroke patients covered all aspects of life, among which information needs, emotional and fatigue management needs, and return to work were daily needs of patients (32). Healthcare professionals need to pay attention to the multiple rehabilitation needs of stroke patients and strengthen the popularization of stroke-related knowledge and rehabilitation knowledge. For example, according to patients’ different needs, develop personalized rehabilitation guidance manuals or modular rehabilitation guidance courses based on different needs to help patients gradually address different types of rehabilitation needs, reduce the learning burden and psychological pressure of PSF patients, and avoid exacerbating fatigue in patients caused by teaching too much knowledge at once. At the same time, PSF patients need to strengthen communication with family members, friends, and peers to gain a sense of belonging in intimate relationships, thereby returning to society as soon as possible. To enhance PSF management, hospitals should integrate fatigue-related guidance into standardized discharge instructions with electronic reminders for follow-up inquiries, establish a tripartite communication mechanism among patients, caregivers, and professionals. For caregivers, concise PSF education leaflets or short videos should be developed, covering the definition of PSF, common manifestations, clarification of misconceptions, and supportive techniques. For healthcare professionals, PSF recognition and management should be included in continuing education modules, and case-based discussions should be used to improve clinical sensitivity. Furthermore, stroke rehabilitation peer support groups could be established, with trained survivors acting as “peer mentors” and organising regular online/offline activities to meet patients’ need for social belonging.

### 4.4 Fatigue symptom management strategies for PSF patients were mainly self-management; healthcare professionals need to provide professional guidance in combination with patients’ opinions

The results of this study show that fatigue management strategies for PSF patients mainly included self-management strategies such as rest and sleep, exercise, planning, and pacing. The reasons may be as follows: patients lacked professional guidance and needed to explore methods to manage fatigue symptoms during symptom experience; and the experience of PSF patients was highly subjective, patients had difficulty describing their own unpleasant symptom experiences, cannot accurately express their needs, and thus struggled to obtain targeted support and help.

Existing studies have shown that non-pharmacological interventions, such as exercise therapy (33) and breathing therapy (34), have a positive impact on alleviating fatigue symptoms in patients with PSF, which is consistent with the self-management strategies employed by these patients. The planning and pacing strategies were also recognized by both patients and healthcare providers (22). Health education on fatigue-related knowledge and self-management for patients with PSF was a key component of management (3). However, there was still no consensus between healthcare professionals and patients on the timing of professional guidance (22), resulting in an information gap when patients seek professional advice.

Adopting individualized and multidisciplinary management plans is crucial for improving the quality of life and treatment effects of PSF patients (3). To further promote symptom management in patients, healthcare providers should communicate with patients by understanding how PSF patients cope with fatigue in daily life, the strategies they use to manage fatigue, and which strategies patients believe are most effective for self-management of their symptoms. When developing fatigue management plans, healthcare professionals should first interview patients to understand their current self-management strategies. A self-management toolkit of PSF should be developed, including fatigue diary templates, activity-rest schedules, relaxation audios, breathing exercise instruction videos, etc., so that patients can choose according to their own situation. What’s more, communication skills training for healthcare professionals should be strengthened to enable patient-centred guidance and avoid information overload or poor timing. And it is necessarily to accelerate the formation of standardized guidelines for PSF symptom management (3) to provide a reference for healthcare providers in professional guidance. In addition, relatives and friends of PSF patients can be encouraged to participate in symptom management actively, play a positive role in managing fatigue symptoms, and help patients reduce their symptom burden.

## 5 Limitations

This study still has limitations. For example, this study only included studies in Chinese and English, and cultural backgrounds may influence the synthesized results. In addition, the quality evaluation of the included literature was generally Grade B, which may have a certain impact on the interpretation of results. What’s more, although this study systematically searched nine Chinese and English databases, some relevant databases were not included in the search. This may have led to the omission of some qualitative studies published in journals or not indexed in the selected databases, potentially affecting the comprehensiveness of the synthesized findings. Future reviews could consider expanding the database coverage, as well as incorporating grey literature searches, hand searching, and reference tracking, to minimise bias arising from incomplete retrieval. Last, the JBI meta-aggregation method employed in this study synthesises the findings of the included original studies and primarily focuses on the aggregation and summation of results rather than in-depth interpretative analysis. Consequently, the synthesised findings largely reflect explicit themes without undertaking theoretical reconstruction. Future research may further incorporate interpretative approaches to enrich the understanding of post-stroke fatigue experiences.

## 6 Conclusion

In summary, this study conducted a meta-synthesis of qualitative studies related to PSF, based on the Theory of Unpleasant Symptoms. The synthesized results indicated that post-stroke fatigue had a multidimensional symptom experience and was affected by multiple factors from physiologic, psychologic, and situational dimensions. There were deficiencies in the cognition of PSF among patients, healthcare professionals, and patients’ relatives and friends; symptom management of PSF was mainly self-management, and patients had multiple rehabilitation needs that urgently need to be met.

It is recommended that subsequent studies develop specific measurement tools for PSF, targeting the multidimensional nature of patients’ fatigue symptom experiences and the factors that influence them. At the same time, caregivers are encouraged to actively participate in patients’ symptom management, provide emotional support, and create a positive and healthy rehabilitation environment. In addition, it is suggested that healthcare professionals conduct popular science activities on stroke and post-stroke fatigue during patients’ hospitalization to enhance stroke patients’ understanding of rehabilitation-related knowledge.

## Data Availability

The original contributions presented in the study are included in the article/Supplementary material, further inquiries can be directed to the corresponding author.
