# Supplementary material for: Meta-synthesis of fatigue symptom experience and influencing factors in post-stroke fatigue patients in a qualitative study
Source: Front Neurol. 2026 Jul 8;17:1763079. doi: 10.3389/fneur.2026.1763079 (PMC13390348; doi:10.3389/fneur.2026.1763079)
Supplement: Supplementary file 1 [file Supplementary_file_1.docx]

| No. | Database | Items found: 614 |
| --- | --- | --- |
| 1 | PubMed | 41 |
| 2 | Web of Science | 210 |
| 3 | Embase | 119 |
| 4 | CINAHL | 140 |
| 5 | Cochrane Library | 60 |
| 6 | CNKI | 13 |
| 7 | Wanfang Data | 2 |
| 8 | VIP Database | 24 |
| 9 | CBM | 5 |

| 1 PubMed search strategies | |
| --- | --- |
| Search terms |  |
| #1 | "stroke"[MeSH Terms] |
| #2 | "Strokes"[Title/Abstract] OR "cerebrovascular accident"[Title/Abstract] OR "cerebral stroke"[Title/Abstract] OR "stroke cerebral"[Title/Abstract] OR "cerebrovascular apoplexy"[Title/Abstract] OR "vascular accident brain"[Title/Abstract] OR "brain vascular accident"[Title/Abstract] OR "cerebrovascular stroke"[Title/Abstract] OR "stroke cerebrovascular"[Title/Abstract] OR "Apoplexy"[Title/Abstract] OR (("Stroke"[MeSH Terms] OR "Stroke"[All Fields] OR "cva"[All Fields]) AND "cerebrovascular accident"[Title/Abstract]) OR "stroke acute"[Title/Abstract] OR "acute stroke"[Title/Abstract] OR "cerebrovascular accident acute"[Title/Abstract] OR "acute cerebrovascular accident"[Title/Abstract] |
| #3 | #1 OR #2 |
| #4 | "post stroke fatigue"[Title/Abstract] OR "Fatigue"[Title/Abstract] OR "PSF"[Title/Abstract] |
| #5 | "psychological"[Title/Abstract] OR "Experience"[Title/Abstract] OR "Needs"[Title/Abstract] OR "perception*"[Title/Abstract] OR "feeling*"[Title/Abstract] |
| #6 | "qualitative research"[Title/Abstract] OR "qualitative study"[Title/Abstract] OR "qualitative methods"[Title/Abstract] OR "Interview"[Title/Abstract] OR "Phenomenon"[Title/Abstract] OR "grounded theory"[Title/Abstract] OR "ethnographic research"[Title/Abstract] |
| #7 | #3 AND #4 AND #5 AND #6 |

| 2 Web of science search strategies | |
| --- | --- |
| Search terms |  |
| #1 | TI=(Stroke OR “cerebrovascular accident” OR Strokes) OR AB=(Stroke OR “cerebrovascular accident” OR Strokes) |
| #2 | TI=(fatigue OR “post stroke fatigue” OR PSF) OR AB=(fatigue OR “post stroke fatigue” OR PSF) |
| #3 | TI=(psychological OR Experience OR Needs OR perception* OR feeling*) OR AB=(psychological OR Experience OR Needs OR perception* OR feeling*) |
| #4 | TI=(“qualitative research” OR “qualitative study” OR “qualitative methods” OR Interview OR Phenomenon OR “grounded theory” OR “ethnographic research”) OR AB=(“qualitative research” OR “qualitative study” OR “qualitative methods” OR Interview OR Phenomenon OR “grounded theory” OR “ethnographic research”) |
| #5 | #1 AND #2 AND #3 AND #4 |

| 3 Embase search strategies | |
| --- | --- |
| Search terms |  |
| #1 | 'stroke'/exp |
| #2 | ‘cerebrovascular accident’:ab,ti OR strokes:ab,ti |
| #3 | fatigue:ab,ti OR ‘post stroke fatigue’:ab,ti OR PSF:ab,ti |
| #4 | psychological:ab,ti OR experience:ab,ti OR Needs:ab,ti OR perception:ab,ti OR feeling:ab,ti |
| #5 | ‘qualitative research’:ab,ti OR ‘qualitative study’:ab,ti OR ‘qualitative methods’:ab,ti OR Interview:ab,ti OR Phenomenon:ab,ti |
| #6 | #2 AND #3 AND #4 AND #5 |

| 4 CINAHL search strategies | |
| --- | --- |
| Search terms |  |
| #1 | XB stroke OR XB “cerebrovascular accident” OR XB strokes |
| #2 | XB fatigue OR XB “post stroke fatigue” OR XB PSF |
| #3 | XB psychological OR XB experience OR XB Needs* OR XB feelings* |
| #4 | XB “qualitative research”l OR XB “qualitative study” OR XB “qualitative methods” OR XB Interview OR XB Phenomenon |
| #5 | #1 AND #2 AND #3 AND #4 |

| 5 Cochrane Library search strategies | |
| --- | --- |
| Search terms |  |
| #1 | (Strokes):ab,ti,kw OR (Cerebrovascular Accident):ab,ti,kw OR (Cerebral Stroke):ab,ti,kw OR (Stroke, Cerebral):ab,ti,kw OR (Cerebrovascular Apoplexy):ab,ti,kw OR (Vascular Accident, Brain):ab,ti,kw OR (Brain Vascular Accident):ab,ti,kw OR (Cerebrovascular Stroke):ab,ti,kw OR (Stroke, Cerebrovascular):ab,ti,kw OR (Apoplexy):ab,ti,kw OR (CVA (Cerebrovascular Accident)):ab,ti,kw OR (Stroke, Acute):ab,ti,kw OR (Acute Stroke):ab,ti,kw OR (Cerebrovascular Accident, Acute):ab,ti,kw OR (Acute Cerebrovascular Accident):ab,ti,kw OR |
| #2 | (post stroke fatigue):ab,ti,kw OR (Fatigue):ab,ti,kw OR (PSF):ab,ti,kw OR |
| #3 | (Psychological):ab,ti,kw OR (Experience):ab,ti,kw OR (Needs):ab,ti,kw OR (perception* ):ab,ti,kw OR (feeling*):ab,ti,kw OR |
| #4 | (qualitative research):ab,ti,kw OR (qualitative study ):ab,ti,kw OR (qualitative methods):ab,ti,kw OR (Interview):ab,ti,kw OR (Phenomenon):ab,ti,kw OR (grounded theory):ab,ti,kw OR (ethnographic research):ab,ti,kw OR |
| #5 | #1 AND #2 AND #3 AND #4 |

| 6 CNKI search strategies | |
| --- | --- |
| Search terms |  |
| #1 | （TKA=脑卒中）OR（TKA=卒中）OR（TKA=中风） |
| #2 | （TKA=疲劳）OR（TKA=脑卒中疲劳）OR（TKA=卒中后疲劳） |
| #3 | （TKA=体验）OR（TKA=经历）OR（TKA=感受） |
| #4 | （TKA=质性研究）OR（TKA=现象学研究） |
| #5 | #1 AND #2 AND #3 AND #4 |

| 7 Wanfang search strategies | |
| --- | --- |
| Search terms |  |
| #1 | [题名或关键词:(脑卒中) or 题名或关键词:(卒中) or 题名或关键词:(中风)](https://s.wanfangdata.com.cn/advanced-search/paper?q=%E9%A2%98%E5%90%8D%E6%88%96%E5%85%B3%E9%94%AE%E8%AF%8D:(%E7%96%B2%E5%8A%B3) or %E9%A2%98%E5%90%8D%E6%88%96%E5%85%B3%E9%94%AE%E8%AF%8D:(%E7%96%B2%E5%80%A6) or %E9%A2%98%E5%90%8D%E6%88%96%E5%85%B3%E9%94%AE%E8%AF%8D:(%E7%96%B2%E4%B9%8F)&searchtype=expert&type=["periodical","thesis","conference"]" \t "https://s.wanfangdata.com.cn/advanced-search/_blank) |
| #2 | [题名或关键词:(疲劳) or 题名或关键词:(脑卒中疲劳) or 题名或关键词:(卒中后疲劳)](https://s.wanfangdata.com.cn/advanced-search/paper?q=%E9%A2%98%E5%90%8D%E6%88%96%E5%85%B3%E9%94%AE%E8%AF%8D:(%E7%96%B2%E5%8A%B3) or %E9%A2%98%E5%90%8D%E6%88%96%E5%85%B3%E9%94%AE%E8%AF%8D:(%E7%96%B2%E5%80%A6) or %E9%A2%98%E5%90%8D%E6%88%96%E5%85%B3%E9%94%AE%E8%AF%8D:(%E7%96%B2%E4%B9%8F)&searchtype=expert&type=["periodical","thesis","conference"]" \t "https://s.wanfangdata.com.cn/advanced-search/_blank) |
| #3 | [题名或关键词:(体验) or 题名或关键词:(经历) or 题名或关键词:(感受)](https://s.wanfangdata.com.cn/advanced-search/paper?q=%E9%A2%98%E5%90%8D%E6%88%96%E5%85%B3%E9%94%AE%E8%AF%8D:(%E7%96%B2%E5%8A%B3) or %E9%A2%98%E5%90%8D%E6%88%96%E5%85%B3%E9%94%AE%E8%AF%8D:(%E7%96%B2%E5%80%A6) or %E9%A2%98%E5%90%8D%E6%88%96%E5%85%B3%E9%94%AE%E8%AF%8D:(%E7%96%B2%E4%B9%8F)&searchtype=expert&type=["periodical","thesis","conference"]" \t "https://s.wanfangdata.com.cn/advanced-search/_blank) |
| #4 | [题名或关键词:(质性研究) or 题名或关键词:(现象学研究)](https://s.wanfangdata.com.cn/advanced-search/paper?q=%E9%A2%98%E5%90%8D%E6%88%96%E5%85%B3%E9%94%AE%E8%AF%8D:(%E7%96%B2%E5%8A%B3) or %E9%A2%98%E5%90%8D%E6%88%96%E5%85%B3%E9%94%AE%E8%AF%8D:(%E7%96%B2%E5%80%A6) or %E9%A2%98%E5%90%8D%E6%88%96%E5%85%B3%E9%94%AE%E8%AF%8D:(%E7%96%B2%E4%B9%8F)&searchtype=expert&type=["periodical","thesis","conference"]" \t "https://s.wanfangdata.com.cn/advanced-search/_blank) |
| #5 | #1 AND #2 AND #3 AND #4 |

| 8 VIP Database search strategies | |
| --- | --- |
| Search terms |  |
| #1 | M=脑卒中 OR M=卒中 OR M=中风 |
| #2 | M=疲劳 OR M=脑卒中疲劳 OR M=卒中后疲劳 |
| #3 | M=体验 OR M=经历 OR M=感受 |
| #4 | M=质性研究 OR M=现象学研究 |
| #5 | #1 AND #2 AND #3 AND #4 |

| 9 CBM search strategies | |
| --- | --- |
| Search terms |  |
| #1 | "脑卒中"[标题:智能] OR "卒中"[标题:智能] OR "中风"[标题:智能] |
| #2 | "脑卒中"[摘要:智能] OR "卒中"[摘要:智能] OR "中风"[摘要:智能] |
| #3 | #1 OR #2 |
| #4 | "疲劳"[标题:智能] OR "脑卒中疲劳"[标题:智能] OR "卒中后疲劳"[标题:智能] |
| #5 | "疲劳"[摘要:智能] OR "脑卒中疲劳"[摘要:智能] OR "卒中后疲劳"[摘要:智能] |
| #6 | #4 OR #5 |
| #7 | "体验"[标题:智能] OR "经历"[标题:智能] OR "感受"[标题:智能] |
| #8 | "体验"[摘要:智能] OR "经历"[摘要:智能] OR "感受"[摘要:智能] |
| #9 | "质性研究"[标题:智能] OR "现象学研究"[标题:智能] |
| #10 | "质性研究"[摘要:智能] OR "现象学研究"[摘要:智能] |
| #11 | #9 OR #10 |
| #12 | #3 AND #6 AND #11 |
